# Awareness and utilization of pre-exposure prophylaxis and HIV prevention services among transgender and non-binary adolescent and young adults

**DOI:** 10.3389/frph.2023.1150370

**Published:** 2024-01-22

**Authors:** Arianna Rodriguez, Keith J. Horvath, Nadia Dowshen, Raina Voss, Jonathan Warus, Megan Jacobs, Kacie M. Kidd, David J. Inwards-Breland, Jill Blumenthal

**Affiliations:** ^1^ Department of Medicine, University of California San Diego, La Jolla, CA, United States; ^2^ Department of Psychology, San Diego State University, San Diego, CA, United States; ^3^ Craig-Dalsimer Division of Adolescent Medicine, Children’s Hospital of Philadelphia, Philadelphia, PA, United States; ^4^ Department of Pediatrics, Perelman School of Medicine, University of Pennsylvania, Philadelphia, PA, United States; ^5^ Division of Adolescent Medicine, Ann & Robert H. Lurie Children’s Hospital of Chicago, Chicago, IL, United States; ^6^ Department of Pediatrics, Children’s Hospital Los Angeles, Keck School of Medicine of USC, Los Angeles, CA, United States; ^7^ Department of Pediatrics, Oregon Health and Science University, Portland, OR, United States; ^8^ Division of Adolescent Medicine, WVU Medicine Children’s, Morgantown, WV, United States; ^9^ Department of Pediatrics, Rady Children’s Hospital, UCSD, La Jolla, CA, United States

**Keywords:** pre-exposure prophylaxis, transgender, non-binary, adolescents, awareness, young adults

## Abstract

**Introduction:**

Transgender and gender non-binary (TGNB) individuals are disproportionally affected by HIV and face high rates of discrimination and stigmatization, resulting in limited access to HIV prevention services. Pre-exposure prophylaxis (PrEP) is highly efficacious for reducing the risk of HIV transmission. However, little research is available regarding PrEP awareness and utilization among TGNB adolescents and young adults (AYA).

**Methods:**

TGNB AYA ages 15–24 years old were recruited between December 2021 and November 2022 for participation in a one-time, anonymous online survey study to assess PrEP awareness and perceptions, as well as barriers to its use. Participants were recruited from seven academic centers offering gender-affirming care to TGNB AYA across the United States.

**Results:**

Of the 156 TGNB AYA individuals who completed the survey, most (67%) were aware of PrEP; however, few (7%) had been prescribed PrEP. Many (60%) had not spoken to a medical provider and, even if the medication was free and obtained confidentially, most participants did not plan to take PrEP due to low perceived HIV risk, lack of PrEP knowledge, and concern about interactions between their hormone therapy and PrEP.

**Discussion:**

These findings underscore the need for broad PrEP educational efforts for both TGNB AYA and their providers to improve knowledge, identify potential PrEP candidates among TGNB AYA and improve access by addressing identified barriers.

## Introduction

Transgender and gender non-binary (TGNB) individuals are disproportionally affected by HIV (1). In 2017, transgender individuals were 3 times more likely than the national average to have a new HIV diagnosis (2), with HIV prevalence 19 times higher for transgender women compared to cisgender women ages 15–49 years old (3). Among all adults and adolescents, 92% of diagnoses of HIV infections in transgender individuals were among transgender women (4). Approximately 1 in 7 (14%) transgender women in the US are estimated to be living with HIV (5).

Disparities in HIV in the TGNB community are even more pronounced within ethnic and racial minority sub-populations. In 2018, 40% of new HIV diagnoses in transgender men and 49% of new HIV diagnoses in transgender women were among Black individuals, higher than any other racial/ethnic group (6). Although prevalence data specific to adolescent transgender individuals of color are limited, recent HIV rates from a meta-analysis of adults estimated 44% of Black transgender women and 26% of Hispanic/Latina transgender women are living with HIV (5).

In order to decrease the incidence of new HIV infection in adolescents and young adults (AYA), an emphasis on effective HIV prevention strategies is essential. Pre-exposure prophylaxis (PrEP) is a highly effective medication for reducing the risk of acquiring HIV when taken consistently and is recommended for individuals at increased risk for infection (7). PrEP has been well studied and FDA approved in adolescents (8, 9). A 2019 systematic review of 14 randomized control trials, 8 observational studies, and 7 studies of diagnostic accuracy demonstrated a 75% reduction in risk of HIV infection comparing PrEP with placebo when there was a high adherence to PrEP (7). However, while more than 20% of new HIV infections occur among AYA, this population accounted for less than 10% of all PrEP prescriptions in 2017 (10).

Despite their higher need for HIV prevention services, little research is available regarding PrEP awareness and engagement among TGNB AYA, although preliminary research has identified some of the barriers to PrEP care. A recent study of 202 TGNB AYA aged 15–24 found an overall high level of PrEP awareness, but little specific knowledge regarding PrEP. While only 0.5% of respondents were using PrEP, over half of those who had heard of PrEP prior to the study were willing to use it (11). Other studies have shown that many sexual and gender minority adolescents assigned male at birth are not aware of PrEP, and many do not know how they could access PrEP if needed (12). A focus group study of adult TGNB participants identified limited healthcare access, mental health problems, potential drug interactions with gender-affirming hormone therapy (GAHT), and lack of protection against sexually transmitted infections (STIs) as barriers to starting PrEP (13). It is also known that TGNB individuals face disproportionally higher rates of discrimination and stigma in general as well as within medicine, which has resulted in limited use of medical services, including HIV prevention (1).

The PrEP care continuum provides a framework for the multiple steps needed to maintain awareness, uptake, adherence, and retention in care (14). In this study, we evaluated PrEP awareness, utilization and preferences as well as perceived barriers to PrEP among TGNB AYAs who seek care in a gender-affirming care clinic.

The primary objective of this study was to evaluate PrEP awareness and current utilization among TGNB adolescents and young adults. The secondary objectives of this study were to assess PrEP preferences among TGNB adolescents and young adults and to determine perceived barriers to PrEP usage among TGNB adolescents and young adults.

## Methods

AYA ages 15–24 years old were recruited for participation in a one-time, anonymous survey to assess engagement in the PrEP continuum of care (including PrEP awareness, use, and attitudes), as well as barriers to its use. Participants were recruited from seven gender-affirming care clinics in the United States at the following institutions: University of California San Diego, Rady Children's Hospital, Children's Hospital of Philadelphia, Lurie Children's Hospital of Chicago, Children's Hospital Los Angeles, Oregon Health and Science University Hospital, and West Virginia University Medicine Children's Hospital. This study recruited from seven clinics in five states housed within academic medical centers in the United States. Clinic sizes ranged from 1 to 15 providers and all routinely prescribed PrEP. Patient population size ranged from 400 to 3,500 patients, and the majority served a predominantly urban population with one clinic serving a rural population. All participating gender-affirming care clinics provided PrEP. To be eligible to participate in the study, individuals needed to be: 15–24 years old, self-identified as transgender or non-binary, English-speaking, and receiving care in a gender-affirming clinic. TGNB youth were passively recruited between December 2021 and November 2022 using flyers posted in waiting rooms and other clinical spaces. Participants scanned a QR code on the flyer using their smart phone and were directed to the online consent form and survey.

The online survey was created using questions adapted from a randomized control trial specific to transgender AYA (15). The questions were initially developed by a measures harmonization group at the Adolescent Medicine Trials Network for HIV Intervention and adapted to fit the needs of our patient population by adjusting the language within the question and shortening the answer choice options to allow for faster completion of survey. The survey contained 26 items, including questions assessing sociodemographic factors, hormone therapy use, sexual risk behaviors and STI history, PrEP awareness, PrEP use and accessibility, perceptions of PrEP, PrEP preferences, willingness to use PrEP, and barriers to use (Supplementary Material). Questions 1 through 11 assessed sociodemographic factors including: age, location, sex assigned at birth, gender identity, ethnicity, race, primary language, highest level of education, employment status, housing status, and insurance status. To assess GAHT usage participants were asked a dichotomous yes/no question: “Are you currently on hormone therapy as part of gender-affirming care?”. If the respondent answered yes, they were diverted to a multiple-choice question to assess the type of hormone therapy. We then asked participants “If yes to the previous question, how do you take hormone therapy?” and they were given the options: pills, injectables, gels, patches, and other. To assess sexual risk behaviors and STI history participants were asked 5 dichotomous yes/no questions with the option to also choose “prefer not to disclose”. Sexual activity was defined as penetrative anal, penetrative vaginal, or oral sex in the last 12 months.

The remainder of the survey was considered “Section 2”; which started with an informative description of PrEP (see Supplementary Material). To asses PrEP awareness, participants were asked “Before today, have you ever heard of people regularly taking anti-HIV medicines BEFORE a sexual or drug use exposure, to reduce the risk of getting HIV? This is called pre-exposure prophylaxis, or PrEP.” Responses were obtained on a likert scale with 1–5 responses ranging from “No, I’ve never heard of it before today” to “Yes, I know a lot about it.” and participants were given the option to “decline to answer”. If participants chose any answer choice that started with “Yes, […]” or “decline to answer” they were directed to questions 17 and 18 which assessed PrEP accessibility and use. This was assessed by asking the following multiple-choice questions “In the past 3 months have you talked to a medical provider about starting PrEP?” and “Have you ever been prescribed PrEP by a healthcare provider? (regardless of whether you took it or not)”. Responses were on a likert scale with 1–5 responses “Yes, and we both thought it was right for me and I should start Prep” to “No, I have never spoken to a provider about starting PrEP.” For the latter responses we used multiple choice and included “Yes, I am on PrEP right now”, “Yes, I was in the past, but I’m not on PrEP anymore”, and “No, I’ve never been prescribed PrEP”. If a participant indicated they had been on PrEP in the past they were directed to question 20. This question assessed accessibility to PrEP by asking the following multiple choice question [select all that apply]: “Did you get your PrEP from the following people or places?”. The options included “Doctor or other health care provider”, “Sex partner”, “Friend”, “Relative”, “Acquaintance”, “Internet”, “Decline to answer”, and a short answer option. If a participant's response indicated they are not currently on PrEP they were directed to complete the remainder of the survey, questions 19, 21–25 assessed willingness to use PrEP, accessibility, and reasons for PrEP non-use via likert scale. Question 19 was “Do you think PrEP is right for you?”. Responses were on a 5-point likert scale from “Yes, PrEP is definitely right for me” to “No, PrEP is definitely not right for me”. Questions 21–24 were as follows: “How likely would you be to take PrEP if you could get it for free?”, “How likely would you be to take PrEP if you could get it for free and without your parent's knowing?”, “Imagine you were interested in starting PrEP. Do you know of a medical provider that would prescribe PrEP to you?”, and “PrEP is currently available with a prescription and is offered free of cost by most insurance companies. Do you plan to start taking PrEP?”. All questions were assessed using a 5-point likert scale from “I would definitely take it” to “I would definitely not take it”. Question 25 asked participants to specify which route of administration of PrEP they would be interested in using: “PrEP is currently only approved for taking one pill every day. However, there may be other ways you can take PrEP in the future that are also effective at preventing HIV. How would you prefer to take PrEP if other options were available?”. Multiple choice answer options included: “As a pill I take every day”, “As an on-demand pill (around when I have sex–that is, 2 pills 2–24 h before sex, 1 pill 24 h after the first dose, and 1 pill 24 h after the second dose)”, “As an injection that I get from my provider every 2 months”, “As an injection that I get from my provider every 6 months”, and “As an implant that is placed under my skin once a year”.

The final survey question assessed reasons for PrEP non-use, participants were asked “What are your reasons for not starting PrEP?” Participants were given 21 different reasons one might not start PrEP and instructed to identify up to three reasons they have not started PrEP. This extensive list was generated based on prior studies of adolescents in parallel age range, including data from TGNB and MSM individuals (11, 15–17).

Participants were de-identified, and once they completed the survey, they were emailed a $10 electronic gift card.

We reviewed completed surveys to identify possible repeat participation by the same participant based on IP addresses, duration to complete the survey, and similar responses. Those that were concerning for duplicates were excluded. We determined the minimal plausible amount of time for survey completion by participants was 1.5 min given the survey was 26 questions and 50% of participants took over 5 min to complete the survey; we concluded that participants who spent less than 1.5 min to complete survey would be omitted from the results. IP addresses were used to minimize fraudulent responses, but allowing one IP address to be utilized twice if it was linked to two separate email addresses and met all other criteria. This was allowed in the event two different participants used the same device to complete the survey. Straightlining is the tendency of survey respondents to select the same response multiple times in a line of answers on a survey. To avoid straightlining participants were presented with one question per page, they were required to answer each question prior to proceeding, and matrix grid questions were avoided wherever possible. Based on these criteria, 22 survey responses were omitted from the study.

Quantitative analysis examined the differences in PrEP awareness and preferences. The data analysis for this study was performed using Qualtrics software, Version XM. Nonparametric test of proportion, the independent Chi-squared test was used, with the level of significance set at 0.05.

The study was approved by the University of California San Diego Institutional Review Board. Parental consent was not required for minors given the low-risk nature of the study.

## Results

### Participant demographics

We received 178 submissions and excluded 22 of these as possible fraudulent responses. Of the 156 valid submissions, the mean age of participants was 18 years old (SD = 2.8). One-hundred and eighteen (76%) were assigned female at birth and 62 (40%) participants identified as male. Fifty-two (67%) identified as non-Hispanic/Latinx and 79 (51%) were White non-Hispanic. Most participants were in high school (51.3%) and had stable housing (82.1%). Just over half (51%) were currently on gender-affirming hormone therapy (GAHT). Regarding their sexual history, 70 (45%) participants had been sexually active within the last 12 months, and 95% had never been diagnosed with an STI. Thirty-three percent of 15–19 year olds had oral sex in the last 12 months and 21% had vaginal sex in the past 12 months. The majority (70%) had never been tested for HIV (Table 1).

**Table 1:** Participants demographics, GAHT Use and sexual health history.

Demographics (*n* = 156)	*N*	%
Age (mean, standard deviation)	18.2, 2.8	
Location by state		
California	76	49
Illinois	47	30
Pennsylvania	14	9
West Virginia	6	4
Oregon	5	3
Other^a^	8	5
Gender identity		
Male	62	40
Female	40	26
Non-binary/third gender	33	21
Other^b^	21	13
Ethnicity		
Hispanic/Latinx	47	30
Race		
White non-Hispanic/Latinx	79	51
Primary Language		
English	151	97
Highest level of education		
High school	102	69
College	46	22
Graduate school	3	8
Prefer not to say	1	1
Employment status		
Full time	19	12
Part/occasionally	49	31
Unemployed	78	50
Unable to work disabled	4	3
Prefer not to say	6	4
Insurance status		
Public	46	30
Private	87	56
Military	3	2
Not sure	20	12
Housing status (In the past 6 months, have spent at least one night in the following)^c^		
Temporarily staying with friend/family	13	8
In a public place not intended for sleeping	1	1
On the street or outside	1.9	3
In a temporary housing program	4	3
Hospital, SNF, hospice	14	9
Have not spent a night in any of the above places	128	82
History of oral sex		
Yes	66	42
History of anal sex		
Yes	22	14
History of vaginal sex		
Yes	41	26
Sexually Active in the last 12 months		
Yes	70	45
History of STI		
Yes	8	5
Currently on hormone therapy?		
Yes	80	51
How do you take hormone therapy? (*N* = 80)		
Pills	16	20
Injectables	48	61
Gels	12	15
Patches	1	1
Others	2	3

^a^ Other included: Indiana, New Jersey, Alabama, Massachusetts, Washington, Ohio.

^b^ Other included: Genderqueer, Gender fluid, Two spirit, Prefer to self-describe, Prefer not to say.

^c^ Zero participants reported that they lived in a shelter, in a welfare voucher hotel/motel, in jail, prison, or halfway house, in drug treatment.

### Prep awareness

104 participants (67%) had heard of PrEP prior to completing the survey. Of the 70 participants who had been sexually active in the last 12 months, 69% had previously heard of PrEP. Participants who identified as non-Hispanic/Latino were more likely to have heard of PrEP compared to Hispanic/Latino individuals, (73% vs. 53%, *p = 0.016*) (Table 2). There were no differences in PrEP awareness by age, gender identity, geographic region, insurance status or sexual activity (Table 2).

**Table 2:** Correlates of PrEP awareness.

	Have you ever heard of PrEP? (*n* = 154)^a^		
	Yes, *n* (%)	No, *n* (%)	*p* ^c^
Age (*n* = 154)			0.073
15–19 years old	71 (68)	41 (82)	
20–25 years old	33 (32)	9 (18)	
Gender Identity (*n* = 154)			0.991
Male	42 (40)	20 (40)	
Female	26 (25)	13 (26)	
Other	36 (35)	17 (34)	
Ethnicity (*n* = 154)			0.016
Hispanic or Latinx	24 (23)	21 (42)	
Non-Hispanic or Latinx	80 (77)	29 (58)	
Race (multiple choice question)			0.170
White, *n*	68	28	
Black, *n*	6	5	
Asian/Pacific Islander/Native Hawaiian, *n*	5	1	
American Indian/Alaskan Native, *n*	2	4	
Mixed Race, *n*	15	10	
Other/Decline to answer, *n*	8	12	
Location/Region in United States (*n* = 154)			0.061
Northwest	7 (7)	0 (0)	
Southwest	56 (53)	20 (40)	
Midwest	28 (27)	19 (38)	
Northeast	12 (12)	11 (22)	
Southeast	1 (1)	0 (0)	
Insurance status (*n* = 136)^b^			0.277
Public	30 (32)	16 (38)	
Private	63 (67)	24 (57)	
Military	1 (1)	2 (5)	
Sexually Active (vaginal) (*n* = 152)			0.760
Yes	27 (26)	14 (29)	
No	76 (74)	35 (71)	
Sexually Active (anal) (*n* = 151)			0.575
Yes	16 (16)	6 (12)	
No	86 (84)	43 (88)	

^a^ 154 participants were included in this analysis as 2 participants declined to answer this question.

^b^ 136 participants were included in this analysis as 20 participants who reported “do not know” were excluded from this analysis.

^c^ *p* values < 0.05 were considered significant. Determined by independent chi-squared analysis.

### Prep access and use

Of those participants who had heard of PrEP prior to the study, only 12% had spoken to a medical provider about starting PrEP in the past 3 months, 3% had been prescribed PrEP in the past and 4% were currently taking PrEP. Of the participants who had a history of sexual activity, 17% had spoken to a healthcare provider about PrEP. Of the 7 patients who had previously or were currently taking PrEP, 5 received PrEP from a health care provider, 1 participant received PrEP from a relative and 1 obtained PrEP from the internet. Of the 99 participants who had never been prescribed PrEP, most (61%) reported knowing of a medical provider that would prescribe PrEP to them.

### Prep perceptions and preferences

Participants not currently on PrEP were asked if they thought PrEP was right for them. Seventy-eight (51%) reported they were not sure, and only 5 (3%) thought PrEP was right for them. When asked if they would take PrEP if they could get it for free, 54 (36%) reported they would take it; when asked if they would take PrEP if it was free and without their parent's knowledge this increased to 66 (44%). Of the participants who did not think they needed PrEP, nearly half (47%) reported a history of sexual activity in the past 12 months, 4% had a history of STI diagnosis in their lifetime, and 32% had been tested for HIV in their lifetime. There were no statistically significant differences in the preference for route of administration of PrEP based on type of GAHT used based on chi-squared test of independence. Participants on both oral (11/14, 79%) and injectable (29/42, 69%) GAHT preferring to take oral PrEP (See Table 3).

**Table 3:** Hormone therapy and route preference of PrEP administration.

	Preference for route of PrEP administration		
	Oral (pill) *n*(%)	Injectable *n*(%)	*p*-value^a^
Current hormone therapy route			0.495
Oral (pill)	11 (28)	3 (19)	
Injectable	29 (72)	13 (81)	

^a^ *p* value < 0.05 was considered significant. Determined by independent chi-squared analysis.

### Willingness and barriers to use PrEP

**Figure 1:**
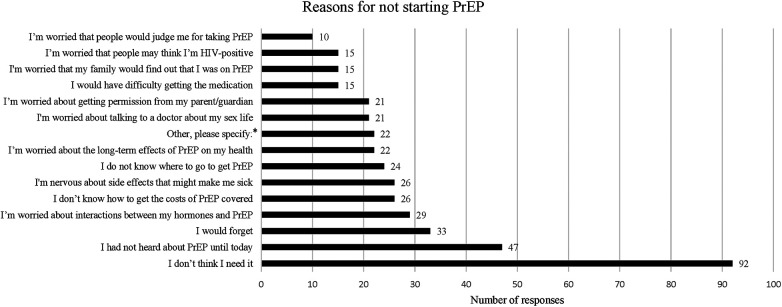
Reasons for not starting PrEP. This was a multiple choice question within the survey, asking participants “What are your reasons for not starting PrEP?”. Participants were given 21 answer options including one answer choice option “Other, please specify:” which allowed for a free text response. Only answers with more than 10 responses are shown in this graph.

When asked if they planned to start taking PrEP, 15 (10%) participants reported they were planning to start taking PrEP. The most frequently cited reason for not starting PrEP endorsed by 92 (61%) participants was not thinking they needed it. Other common reasons for not starting PrEP included not being aware of PrEP previously, worries about forgetting to take it, interactions with GAHT, financial coverage, side effects, not knowing where to get PrEP, long term effects of PrEP, needing parental permission, and stigma (See Figure 1).

## Discussion

This anonymous online survey study suggests that the majority of TGNB AYA who are followed at gender-affirming health clinics are aware of PrEP; however, few had been prescribed or were currently taking PrEP. These findings are similar to prior studies regarding PrEP awareness and use in TGNB AYA (11, 18, 19).

Many participants remained hesitant to start PrEP, with the main reason being they did not think they needed it. Our data showed just under half of the participants who did not think they needed PrEP reported a history of sexual activity in the last three months; however, this information alone does not necessarily place these patients at higher risk for HIV and we are unable to postulate the HIV risk in this sample. To determine if a patient should be started on PrEP it would be relevant to obtain information on barrier contraception, if they have been diagnosed with an STI in the past 6 months, HIV status of partners, injection drug use history, and post-exposure prophylaxis use which were not obtained in this study. This finding does underscore the importance of speaking with AYA confidentially and obtaining a sexual history, discussing sexual health behaviors that are associated with the risk of HIV acquisition, and introducing the topic of PrEP as recommended by the CDC for all sexually active individuals (5, 20, 21). Further, of participants who had never been prescribed PrEP, nearly 40% reported not knowing of a medical provider to prescribe them PrEP which is relevant given all gender-affirming health clinics who participated in the survey offering PrEP. These findings together emphasize the need for patient and healthcare provider education as well as provider responsibility to initiate these conversations.

TGNB AYA who identified as Hispanic/Latinx were less likely to be aware of PrEP than their non-Hispanic/Latinx counterparts, consistent with the 2019 National HIV Prevention Program Monitoring and Evaluation HIV testing data which showed that PrEP awareness among Hispanic persons was lower compared with non-Hispanic persons (22). Expanding culturally appropriate PrEP educational efforts is a key step to eliminating this disparity and reducing the impact of HIV on this community (23). Research studies encompassing a larger Hispanic/Latinx population are critical to better understanding how to specifically address these topics in this community.

Participants also noted not starting PrEP due to financial concern. Most of this patient population had private or public health insurance; while many insurers may cover PrEP it is unclear what co-pay the respondent may be responsible for and this may still pose a financial barrier to the respondent. Furthermore, given the age range of participants, some may have been on their parents' insurance plan which may require disclosure to a parent to obtain. Although few individuals reported they would take PrEP, many said they would take it if it was free and even more would take PrEP if it was free and without their parent's knowledge. Similar concerns have been reported in prior studies of cisgender sexual minority adolescents (12). This increase in willingness to take PrEP suggests that many individuals are interested in taking PrEP, but financial and confidentiality-related barriers may be preventing them from starting the medication. Nearly all public and private insurers cover PrEP. In instances where a patient is uninsured or whose insurance does not cover PrEP medication, there are programs available to provide PrEP for free; however most are only available to adults (21). Financial counseling is an important step to ensure successful PrEP uptake, persistence, and preventing barriers to care. While PrEP has been FDA approved for adolescents, each state's minor consent laws may impact access for adolescents including being able to confidentially obtain PrEP (24, 25).

Similar to what has been found in other studies (11), participants were concerned about the possibility of drug interactions with their GAHT. This hesitation to start PrEP would require a multimodal approach to impact behavioral change in this population. Educating AYA on studies that have shown PrEP medications do not impact the effect of GAHT could be an important way to begin this conversation (26–29).

Most participants preferred to take PrEP as an oral daily tablet over a longer acting injectable/implant or on demand tablet, which was a surprising finding given many used injectable GAHT. Among other variables, it is possible that they were more familiar with oral PrEP given it was the only FDA approved option prior to December 2021. Within one study, interviews among trans women on GAHT revealed they were concerned that they would need to be physically present in clinic to receive injectable PrEP and would only want to use injectable PrEP if they could inject it themselves (21).

Limitations of this study include that it may not represent all TGNB AYA given the relatively small sample size. The surveys were only distributed at pediatric gender health clinics affiliated with large university health systems and thus focused on individuals with reasonable access to medical care and potentially higher health literacy compared to individuals not being followed by a physician in a gender health clinic. Individuals were required to have access to a smart phone to complete the survey, and it was only available in English. In addition, the population's demographics differed in our study compared to those of the target population based on the 2014 CDC's Behavioral Risk Factor Surveillance System (BRFSS) (30). The percentage of Black (non-Hispanic) individuals in our sample (4%) is notably lower than the estimated target population from the BRFSS (15%). A significant limitation given the potentially unique experiences of this population. Although convenience sample data have contributed significantly to our understanding of the transgender population, these differences highlight its potential biases. The underrepresentation of black participants in our survey study can be attributed to a combination of factors including institutional and structural racism, geographic distribution of our clinics, and socio-economic factors affecting access to gender-affirming care. Future research engaging patients outside of university affiliated gender health clinics could aid in reaching these populations and should explore more inclusive recruitment strategies to address these limitations; including culturally congruent research processes, benefits of participation, and altruism toward and involvement of family or community (31).

Our survey was distributed at gender health centers in large metropolitan regions notably located in states with less restrictive laws on access to gender affirming care; thus many states with more restrictive laws are not represented in this sample. We acknowledge that while strategies were utilized to minimize fraudulent responses there were other ways in which fraudulent responses could have been missed and still included in the study given our inability to use advanced technology to stop a participant from completing repeated attempts of survey from different IP addresses. This method was not used due to funding limitations. Given that many of the questions were based on the same rating scale, there could have been some non-differentiation in ratings that contributed to fraudulent responses. Due to limitations in the duration of the study and prioritization of high value questions to avoid survey fatigue, we did not obtain detailed information regarding facilitators of PrEP uptake as well as specific HIV risk such as barrier precautions, history of STIs and partner status to determine the HIV risk of each individual. Future research should specifically include this valuable information.

Our study underscores the need for broad PrEP educational efforts for both TGNB AYA and their healthcare providers to improve knowledge, identify potential PrEP candidates among TGNB AYA at risk for HIV infection and improve access by addressing identified barriers. Many of the reasons obtained in this study for not taking PrEP are common misconceptions which can be addressed with AYA during medical visits. These findings should inform clinical practice for healthcare providers with an emphasis on ensuring patients are aware of PrEP, helping them obtain PrEP, and addressing common concerns for not starting PrEP. Future studies examining reasons for slow uptake of PrEP as well as engaging Black and Latinx TGNB AYA to develop interventions for PrEP engagement are needed.

## Data Availability

The raw data supporting the conclusions of this article will be made available by the authors, without undue reservation.
