# Supplementary material for: Awareness and utilization of pre-exposure prophylaxis and HIV prevention services among transgender and non-binary adolescent and young adults
Source: Front Reprod Health. 2024 Jan 22;5:1150370. doi: 10.3389/frph.2023.1150370 (PMC10839107; doi:10.3389/frph.2023.1150370)
Supplement: Supplementary file 1 [file Table1.docx]

Supplementary Material

Awareness and Utilization of Pre-exposure Prophylaxis and HIV Prevention Services Among Transgender and Non-binary Adolescent and Young Adults

**Arianna Rodriguez MD**^1^**, Keith J. Horvath PhD**^2^**, Nadia Dowshen MD MSHP**^3,4^**, Raina Voss MD MPH**^5^**, Jonathan Warus MD**^6^**, Megan Jacobs MD**^7^**, Kacie M. Kidd MD**^8^**, David J. Inwards-Breland MD MPH**^9,10^**†, Jill Blumenthal MD MAS**^1^**†**

*** Correspondence:** Arianna Rodriguez MD: [amr002@health.ucsd.edu](mailto:amr002@health.ucsd.edu)

**Supplementary Material: Survey Questions**

**Section 1: Demographics and Risk**

1. What is your age?

- _ _

1. In what state do you reside?

- _____________

1. What sex were you assigned at birth, on your original birth certificate?

- Male
- Female
- Intersex

1. How do you describe your gender Identity?

- Male
- Female
- Non-binary
- Genderqueer
- Gender fluid
- Two spirit
- Prefer to self-describe__________
- Prefer not to say

1. Ethnicity

- Check only one category
- Hispanic or Latino
- Not Hispanic or Latino
- Does not want to report
- Does not know

1. Race, *Check all that apply*

- American Indian/Alaskan Native
- Asian
- Native Hawaiian or other Pacific Islander
- Black or African American
- White
- Other
- Does not want to report
- Does not know

1. Primary Language

- English
- Spanish
- Other

1. Highest Level of Education

- Some high school
- In high school
- Completed high school
- In college
- Completed college
- In graduate school
- Completed graduate school
- Refused to answer
- Other __________

1. Employment Status

- Full time
- Part time/Occasionally
- Unemployed
- Unable to work (disabled)
- Refused to answer

1. Housing Status: In the past 6 months, have you spent at least one night (*Check all that apply*):

- In a shelter?
- In a public place not intended for sleeping (e.g., bus station, car, abandoned building)?
- On the street or anywhere outside (e.g., park, sidewalk)?
- Temporarily doubled up with a friend or family member?
- In a temporary housing program?
- In a welfare or voucher hotel/motel?
- In jail, prison, or a halfway house?
- In drug treatment, a detox unit, or drug program housing?
- In a hospital, nursing home, or hospice?
- I have not spent a night in any of the above places.

1. Insurance status

- Public (i.e. Medicaid, Medicare, etc.)
- Private
- Military
- None
- Not sure

1. a. Are you currently on hormone therapy as part of gender-affirming care?

- No
- Yes

12. b. If Yes, do you take, *Check all that apply*

- Pills
- Injectables
- Gels
- Patches
- Other

1. Have you had sex in the last 12 months? *[By sex we are referring to either oral (mouth on anus, penis or vagina), anal (penis in anus) or vaginal (penis in vaginal)]?*
   1. Oral
      - No

- Yes
- Prefer not to disclose
  1. Anal
- No
- Yes
- Prefer not to disclose
  1. Vaginal
- No
- Yes
- Prefer not to disclose

1. Have you been ever diagnosed with a sexually transmitted infection? (gonorrhea, chlamydia, syphilis)?

- No
- Yes
- Prefer not to disclose

1. Have you ever been tested for HIV?

- No
- Yes
- Prefer not to disclose

## Section 2: PrEP Measures

### The following questions are about a medication that may be available to you that would lower your chances of becoming infected with HIV.

### PrEP (pre-exposure prophylaxis) is a way to prevent HIV infection. PrEP involves HIV-negative individuals taking anti-HIV medications once a day, every day to reduce the chance of HIV infection if they were exposed to HIV. It is recommended that individuals on PrEP go to a medical provider every 3 months for HIV/STI testing, bloodwork, and a new 3-month prescription.

### Please note that PrEP is not the same as taking HIV medications for a brief period of time (i.e., 28 days) after a high risk exposure to HIV through situations such as being stuck by a contaminated needle or having unprotected sex. PrEP is intended for regular, long-term use.

**Logic: Show/hide trigger exists.**

#### Before today, have you ever heard of people regularly taking anti-HIV medicines BEFORE a sexual or drug use exposure, to reduce the risk of getting HIV? This is called pre-exposure prophylaxis, or PrEP.

( ) No, I’ve never heard of it before today

( ) Yes, I’ve heard about it, but I didn’t really know what it was

( ) Yes, I know a little bit about it

( ) Yes, I know a fair amount about it

( ) Yes, I know a lot about it

( ) Decline to answer

**Logic: Hidden unless: Question "Before today, have you ever heard of people regularly taking anti-HIV medicines BEFORE a sexual or drug use exposure, to reduce the risk of getting HIV? This is called pre-exposure prophylaxis, or PrEP.

 " is one of the following answers ("Yes, I’ve heard about it, but I didn’t really know what it was","Yes, I know a little bit about it","Yes, I know a fair amount about it","Yes, I know a lot about it","Decline to answer")**

#### In the past 3 months have you talked to a medical provider about starting PrEP?

( ) Yes, and we both thought it was right for me and I should start PrEP

( ) Yes, and we both thought it might be right for me but to wait before beginning PrEP

( ) Yes, and we both thought it was not right for me

( ) Yes, and the provider was not comfortable prescribing PrEP for me

( ) Yes, and the provider thought it was right for me but I chose not to do it

( ) No, I have never spoken to a provider about starting PrEP

**Logic: Hidden unless: Question "Before today, have you ever heard of people regularly taking anti-HIV medicines BEFORE a sexual or drug use exposure, to reduce the risk of getting HIV? This is called pre-exposure prophylaxis, or PrEP.

 " is one of the following answers ("Yes, I’ve heard about it, but I didn’t really know what it was","Yes, I know a little bit about it","Yes, I know a fair amount about it","Yes, I know a lot about it")**

#### Have you ever been prescribed PrEP (pre-exposure prophylaxis) by a healthcare provider (regardless of whether you took it or not)?

( ) Yes, I am on PrEP right now

( ) Yes, I was in the past, but I’m not on PrEP anymore

( ) No, I’ve never been prescribed PrEP

**Logic: Show/hide trigger exist. Hidden unless: Question "**Have you ever been prescribed PrEP (pre-exposure prophylaxis) by a healthcare provider (regardless of whether you took it or not)?**"**

**is one of the following answers:** ("Yes, I was in the past, but I’m not on PrEP anymore”, “No, I’ve never been prescribed PrEP)

#### Do you think PrEP is right for you?

( ) Yes, PrEP is definitely right for me.

( ) Yes, I think PrEP is right for me.

( ) I’m not sure if PrEP is right for me.

( ) No, I don’t think PrEP is right for me.

( ) No, PrEP is definitely not right for me.

**Logic: Hidden unless: Question "**Have you ever been prescribed PrEP (pre-exposure prophylaxis) by a healthcare provider (regardless of whether you took it or not)?”
 **" is one of the following answers ("Yes, I am on PrEP right now","Yes, I was in the past, but I’m not on PrEP anymore")**

#### Did you get your PrEP from the following people or places? Please select all that apply.

[ ] Doctor or other health care provider

[ ] Sex partner

[ ] Friend

[ ] Relative

[ ] Acquaintance

[ ] Internet

[ ] Other, please specify: _________________________________________________*

[ ] Decline to answer

**Page entry logic:** This page will show when: Question: **"**Have you ever been prescribed PrEP (pre-exposure prophylaxis) by a healthcare provider (regardless of whether you took it or not)?”

“ is one of the following answers ("Yes, I was in the past, but I’m not on PrEP anymore","No, I’ve never taken PrEP","Decline to answer")

#### How likely would you be to take PrEP if you could get it for free?

( ) I would definitely take it

( ) I would probably take it

( ) I might take it

( ) I would probably not take it

( ) I would definitely not take it

1. How likely would you be to take PrEP if you could get it for free and without your parent’s knowing?

( ) I would definitely take it

( ) I would probably take it

( ) I might take it

( ) I would probably not take it

( ) I would definitely not take it

#### Imagine you were interested in starting PrEP.  Do you know of a medical provider that would prescribe PrEP to you?

( ) Yes, definitely

( ) Yes, probably

( ) I might

( ) No, probably not

( ) No, definitely not

1. PrEP is currently available with a prescription and is offered free of cost by most insurance companies. Do you plan to start taking PrEP?

( ) I would definitely take it

( ) I would probably take it

( ) I might take it

( ) I would probably not take it

( ) I would definitely not take it

1. PrEP is currently only approved for taking one pill every day. However, there may be other ways you can take PrEP in the future that are also effective at preventing HIV. How would you prefer to take PrEP if other options were available?

( ) As a pill I take every day

( ) As an on-demand pill (around when I have sex--that is, 2 pills 2-24 hours before sex, 1 pill 24 hours after the first dose, and 1 pill 24 hours after the second dose)

( ) As an injection that I get from my provider every 2 months

( ) As an injection that I get from my provider every 6 months

( ) As an implant that is placed under my skin once a year)

**Page entry logic:** This page will show when: Question **"**Have you ever been prescribed PrEP (pre-exposure prophylaxis) by a healthcare provider (regardless of whether you took it or not)?”

 " is one of the following answers ("Yes, I was in the past, but I’m not on PrEP anymore","No, I’ve never taken PrEP","Decline to answer")

#### What are your reasons for not starting PrEP? Please select all that apply

[ ] I had not heard about PrEP until today

[ ] My work/school schedule could get in the way

[ ] I would forget

[ ] I don’t think I need it

[ ] I would have difficulty getting the medication

[ ] I don’t know how to get the costs of PrEP covered

[ ] I do not know where to go to get PrEP

[ ] I am worried about talking to a doctor about my sex life

[ ] I tried to access PrEP, but my doctor wouldn’t prescribe it for me

[ ] I am not able to return for PrEP medical check-ups every 3 months

[ ] I’m concerned that PrEP might not provide complete protection against HIV

[ ] I am nervous about side effects that might make me sick

[ ] I’m worried about the long-term effects of PrEP on my health

[ ] I’m worried about interactions between my hormones and PrEP

[ ] My friends or family would not support me taking PrEP

[ ] I’m worried that my friends would find out that I was on PrEP

[ ] I am worried that my family would find out that I was on PrEP

[ ] I’m worried my sexual partners would find out I was on PrEP

[ ] I’m worried that people would judge me for taking PrEP

[ ] I’m worried that people may think I’m HIV-positive if they see that I’m taking HIV medications as PrEP

[ ] I’m worried about getting permission from my parent/guardian

[ ] Other, please specify: _________________________________________________*

[ ] Decline to answer
